# Automation of a problem list using natural language processing

**DOI:** 10.1186/1472-6947-5-30

**Published:** 2005-08-31

**Authors:** Stephane Meystre, Peter J Haug

**Affiliations:** 1 Department of Medical Informatics, University of Utah School of Medicine, Salt Lake City, Utah, USA

## Abstract

**Background:**

The medical problem list is an important part of the electronic medical record in development in our institution. To serve the functions it is designed for, the problem list has to be as accurate and timely as possible. However, the current problem list is usually incomplete and inaccurate, and is often totally unused. To alleviate this issue, we are building an environment where the problem list can be easily and effectively maintained.

**Methods:**

For this project, 80 medical problems were selected for their frequency of use in our future clinical field of evaluation (cardiovascular). We have developed an Automated Problem List system composed of two main components: a background and a foreground application. The background application uses Natural Language Processing (NLP) to harvest potential problem list entries from the list of 80 targeted problems detected in the multiple free-text electronic documents available in our electronic medical record. These *proposed *medical problems drive the foreground application designed for management of the problem list. Within this application, the extracted problems are proposed to the physicians for addition to the official problem list.

**Results:**

The set of 80 targeted medical problems selected for this project covered about 5% of all possible diagnoses coded in ICD-9-CM in our study population (cardiovascular adult inpatients), but about 64% of all instances of these coded diagnoses. The system contains algorithms to detect first document sections, then sentences within these sections, and finally potential problems within the sentences. The initial evaluation of the section and sentence detection algorithms demonstrated a sensitivity and positive predictive value of 100% when detecting sections, and a sensitivity of 89% and a positive predictive value of 94% when detecting sentences.

**Conclusion:**

The global aim of our project is to automate the process of creating and maintaining a problem list for hospitalized patients and thereby help to guarantee the timeliness, accuracy and completeness of this information.

## Background

The problem list is an important piece of the medical record as well as a central component of the problem-oriented electronic medical record in development in our institution (Intermountain Health Care, Utah). To serve the functions it is designed for, the problem list has to be as accurate and timely as possible. In most of our inpatient settings, we are converting to an electronic problem list from a paper-based tool. However, the current problem list, hand written on a form in the paper chart, is usually incomplete and inaccurate, and is often totally unused. While we hope that the advantages of a universally available problem list will change this behavior, as an addition incentive, we are building an environment where the problem list is easily and effectively maintained.

### The medical problem list

More than three decades ago, Larry Weed proposed the Problem-Oriented Medical Record as a remedy for the complexity of the medical knowledge and clinical data, and for weaknesses in the documentation of medical care [1,2]. He noted the lack of consistent structure and content in the progress notes that make up a large part of the medical record. He proposed a standard approach emphasizing a list of patient medical problems that is scrupulously maintained by those caring for the patient. This problem list serves the dual purpose of providing a brief, formal summary of the patient's illnesses and of acting as a tool for organizing the routine documentation of the physician's decision-making process and the plan for and results of care. The problem-oriented, Computer-based Patient Record (CPR) and the problem list have seen renewed interest as an organizational tool in the recent years [3-10], but most of today's patient records retain a time-oriented structure. The Institute of Medicine report on the CPR [11] recommends that it contain a problem list that specifies the patient's medical problems and the status of each. It mentions advantages to this approach: the problem list can be the central place for clinicians to obtain a concise view of all patients' medical problems; this list facilitates associating clinical information in the record to a specific medical problem; and the problem list can encourage an orderly process of medical problem solving and clinical judgment. The problem list in a problem-oriented patient record also provides a context in which continuity of care is supported, preventing both redundant and peripheral actions [3].

### Problem list entries coding

To enable most of these potential advantages, problem list entries in the electronic medical record should be coded, which means that medical problems entered must have a corresponding code in a controlled vocabulary. Medical vocabularies used in problem lists are numerous, ranging from ICD-9-CM [12], to SNOMED [13], the Unified Medical Language System (UMLS^®^) [14-16], and to locally developed vocabularies [17]. SNOMED-CT has been shown to allow 98.5% coverage of problem list terms [13]. Coding of medical problems may be achieved by manually assigning a code when the problem is entered, or by using NLP techniques to map free-text problem list entries with an appropriate code, as described below. The former method is usually eased by the use of pick lists or search engines [18], both features available in our institution's application for managing the problem list. While tools for structured problem entry provide a simple way to assure the availability of coded problems, using NLP techniques to extract coded data from free text allows the use of natural language as the input medium. Natural language remains the most user-friendly and expressive way of recording information, and the application of NLP can still provide the advantages of coded data.

### Medical text mapping to standard vocabularies

Many authors have reported on methods to automatically map clinical text concepts to a standardized vocabulary such as the Unified Medical Language System (UMLS^®^) [19-26]. An example is MetaMap [22,27], an application developed by the US National Library of Medicine (NLM) as an element of the SPECIALIST™ system [28,29]. MetaMap is used to index text or to map concepts in the analyzed text with UMLS concepts. It returns ranked medical concepts, but no negation detection is performed. Five steps are needed, beginning with noun phrase identification using the SPECIALIST minimal commitment parser [30], followed by variant generation, candidate phrase retrieval, and computation of a score for each candidate by comparing it with the input phrase. The process ends with the mapping and the ranking of candidates using the computed score. MetaMap has been shown to identify most concepts present in MEDLINE titles [31]. It has been used for Information Retrieval [32-34], for Information Extraction in biomedical text [31,35], and to extract different types of information like anatomical concepts [36] or molecular binding concepts [37]. It has also been used with electronic messages submitted by patients to automatically provide relevant health information to the patients [24]. Finally, the system was able to extract the most critical findings in 91% of the documents in a previous study done on pathology reports [38].

Another approach to mapping concepts in text to UMLS concepts is found in an application called IndexFinder. IndexFinder functions by generating all valid UMLS concepts by permuting the set of words in the text, and selecting relevant concepts using syntactic and semantic filtering [26].

### Negation detection algorithms

The techniques described above can find medical problems, but they are not designed to detect negation. MetaMap, in particular, does not do negation detection. In the medical domain finding negatives is essential due to the fact that findings and diseases are often described as absent.

Several independent negation detection algorithms have been developed. These include NegEx, a computationally simple algorithm using regular expressions [39], and the more complex general-purpose Negfinder [40]. These algorithms have been evaluated and have shown good results. NegEx has shown a sensitivity of 94.5% and a specificity of 77.8% [39], and Negfinder demonstrated a sensitivity of 95.3% and a specificity of 97.7% [40]. After its evaluation, NegEx was updated to a version 2, available on the main author's website [41].

### Natural language processing in medicine

The patient record contains a considerable amount of information in addition to problem list entries. However, much of the recorded clinical information is unstructured text, also called free-text. These free-text documents represent patient history and reports of therapeutic interventions or clinical progress and make up a substantial part of the medical record. The increasing use of encoded data in decision support and the requirement for standard medical data sets creates a need for coded information instead. As a possible answer to this issue, NLP can convert narrative text into coded data, and therefore extend the use of the CPR [42].

Several groups have evaluated techniques for automatically encoding textual documents from the medical record. The Linguistic String Project [43,44] has developed a series of tools for analyzing medical text. X-ray reports appear to be an especially fertile ground for NLP. Two groups have developed systems whose focus is the radiologist's report of the chest x-ray. Zingmond [45] has applied a semantic encoding tool to these reports to recognize abnormalities that should receive follow-up, and Friedman has studied techniques for encoding interpretations found in these reports [46-50]. In addition, Friedman and her colleagues have studied NLP in mammography reports [51], neuroradiology reports [19], discharge summaries [52], and pathology reports [53]. Good performance was demonstrated. The application developed, called MedLEE (Medical Language Extraction and Encoding system) [54], has also been used to help develop and maintain vocabularies [55], and recently adapted to extract UMLS concepts from medical text documents, achieving 83% recall and 89% precision [56]. MedLEE was also the first biomedical NLP system that was applied to data in an institution different than the one where it was developed. This resulted in a small drop in performance, but after making some adjustments, it performed as well as in the original institution [49].

The understanding of natural language has been a topic of interest for our Medical Informatics group at the LDS Hospital and the University of Utah (Salt Lake City) for a number of years. SPRUS (Special Purpose Radiology Understanding System) [57,58] was the first NLP application developed at the Medical Informatics group at the University of Utah, and was only semantically driven. Later came SymText (Symbolic Text processor) [59-61], with syntactic and probabilistic semantic analysis. The latest version is called MPLUS [62], provides interleaved syntactic analysis based on a context-free grammar with a bottom-up chart parser, interleaved with object-oriented semantic analysis.

SymText and its successor, MPLUS, make extensive use of semantic networks for semantic analysis. These networks are implemented as Bayesian networks (also called belief networks) [63], trained to infer probabilistic relationships between extracted terms and their meaning. This approach has the advantages of being tolerant to noisy data, and of allowing training to refine performances.

A key realm for testing various approaches has been in the Radiology Department. Here we have focused on reports for chest radiographs [58-60] with a further focus on the data in these reports that supports the diagnosis of pneumonia [64,65]. Admit diagnoses [66] and reports describing the results of pulmonary perfusion scans have also been used to test these NLP approaches [67]. For the project described in this paper, MPLUS has been adapted and trained to extract medical problems from electronic free-text documents [68].

### Medical document models

Research into modeling medical documents has been a focus in the development of standards for the electronic medical record. A central example is the first ANSI-approved healthcare standard: the HL7 Clinical Document Architecture (CDA) [69]. It uses XML (eXtensible Markup Language) [70] to facilitate the exchange of documents between users. XML is a data modeling toolkit, a configurable vehicle for any kind of information, and an evolving open standard consistent with a variety of Internet applications. It can store and organize most kinds of data, offers many ways to check the quality of documents, and is easy to read and parse by humans and programs alike.

Several examples of specific medical documents exist. In one case, a successful prototype of a CDA-based, structured discharge summary system was implemented for use in the clinical and community environments of a family practice [71]. In Germany discharge and referral information has been exchanged between Hospital Information Systems and Physician Office Systems in the SCIPHOX project [72]. And in other medical settings this formalism has been used to format discharge letters [73,74].

### Regular expressions

In this project, we have also made heavy use of tools to process regular expressions (sometimes abbreviated as RE, regexp or regex). These are templates that describe a whole set of strings, according to certain syntactic rules. Regular expressions are used by many text editors and utilities to search bodies of text for certain patterns and, for example, replace the found strings with a certain other string. Regular expressions trace back to the work of Stephen Kleene, a mathematician, who described these models using his mathematical notation called "regular sets" [75]. His work was used to develop text-manipulation tools on the Unix platform (including ed [76] and grep) as well as in many other scripting languages, editors, programming environments, and specialized tools such as: expr, awk, Emacs, vim, lex, Perl, Java™ and Microsoft^® ^Word. Regular expressions are well described in many publications [77].

## Methods

### Targeted medical problems

The first step in the process of developing a NLP application to support a problem list was to identify the medical problems for which the system would be responsible. Since the focus of this project has been in the cardiovascular domain, we developed the problem list application with an emphasis on the patient population seen there. Problems appropriately entered into the problem list include both simple observations, representing basic abnormalities noted during a patient's workup (ex: dyspnea, hyperkalemia), and interpretive statements that may represent either syndromic descriptions (ex: Respiratory Failure) or the etiologies for the simple abnormalities that may appear there (ex: Myocardial Infarction as an etiology for Chest Pain). Simple medical problems, whose etiology is not yet understood, are included as they are recognized. Etiologic problems are entered as the physician becomes convinced that they represent a reasonable interpretation of the patient's condition.

For our prototype, we focused on a limited set of 80 medical problems. These problems are listed in Table 1. They were selected for their frequency of use in our institution and in the field of evaluation of our system (cardiovascular). Two sources of information were used to create this list: the log of all coded concepts stored in the central clinical database, called Clinical Data Repository (CDR) in our institution, and a list of the top 25 diagnoses for cardiovascular patients at the LDS Hospital (Salt Lake City, Utah). The first author extracted all concepts coded as medical problems from the log, and ordered them by number of uses during the year 2002. The most common general diagnoses (37 of them) and cardiovascular diagnoses (34 of them) were selected, and 9 of the top 25 cardiovascular diagnoses at the LDS Hospital were added to this list. The resulting list was finally submitted to two expert physicians (a board-certified Internal Medicine specialist and a board-certified Critical Care specialist with more than 20 years of experience) for validation. The second expert is the head of one of the departments where our system will be evaluated at the LDS Hospital.

**Table 1:** List of the selected medical problems. Origin of the problems: 37 from most frequent diagnoses at IHC°, 9 from the field of evaluation (LDS Hospital 7th floor)*, and 34 from other IHC diagnoses (cardiovascular).

Anemia	Hyperlipidemia°
Angina°	Hypertension°
Anxiety°	Hypotension
Aortic stenosis°	Hypothyroidism°
Aortic valve insufficiency	Hypovolemia*
Arrhythmia°	Infectious Endocarditis°
Arthralgias°	Ischemic Heart Disease*
Arthritis°	Left bundle branch block
Asthma°	Left ventricular hypertrophy
Atrial fibrillation°	Melena
Atrial Septal Defects	Mitral stenosis°
Back pain°	Mitral valve insufficiency
Cancer°	Myocardial Infarction°
Cardiac arrest	Obesity°
Cardiogenic Shock°	Pain°
Cardiomyopathy	Paroxysmal supraventric. tachycard.
Cerebrovascular accident°	Peptic Ulcer*
Coma	Pericardial tamponade
Congenital heart disease	Pericarditis*
Constipation°	Peripheral vascular disease°
Coronary artery disease°	Pneumonia*
Deep vein thrombosis	Pneumothorax*
Depression°	Pulmonary edema
Diabetes mellitus°	Pulmonary embolus*
Diplopia°	Pulmonary hypertension
Dysphagia°	Renal insufficiency°
Dyspnea	Restless legs
Emphysema°	Rheumatic heart disease
Epistaxis	Right bundle branch block
Fatigue°	Septicemia
Gastroesophageal reflux°	Sinusitis°
Gout°	Streptococcal sore throat
Headache°	Syncope*
Heart block	Tobacco Use Disorder
Heart failure*	Tricuspid valve insufficiency
Heart Murmur	Urinary tract infection°
Hematemesis	Varicose veins
Hematuria°	Venous insufficiency
Hemoptysis	Ventricular ectopic beats
Hypercholesteremia°	Wheeze

To estimate the proportion of patient's coded diagnoses covered by our set of targeted problems, we retrieved ICD-9-CM codes assigned for administrative purposes to all cardiovascular adult inpatients during 2003 in our institution (LDS Hospital, Salt Lake City, Utah), and compared it with our set of problems. ICD-9-CM codes were selected because they were the only coded medical problems available for cardiovascular inpatients at the LDS Hospital at this time.

### Automated Problem List system

The Automated Problem List (APL) system was designed to extract potential medical problems from free-text medical documents, and uses NLP to achieve this task. It is constructed of two main components: a background application and the problem list management application.

The background application does all text processing and analysis and stores extracted medical problems in the Clinical Data Repository. These medical problems can then be accessed by the problem list management application integrated into our Clinical Information System. Clinicians use this application to access and confirm the proposals of the APL system. The problem list management application allows the viewing, editing and creation of medical problems, and gives access to Internet-based medical knowledge sources allowing rapid review of medical facts for each encoded problem. This latter feature is called "infobutton" [78]. The medical problems extracted by our background application are listed in this application, and link back to a customized view of the source document(s). This display of the document(s) that each problem was extracted from helps users of the problem list determine if a medical problem should be part of the "official" problem list.

### Information model for medical documents and problems

The information model used by our system was created to ease the exchange of data between the background application and the problem list management application. It models medical documents and medical problems.

**Figure 1:**
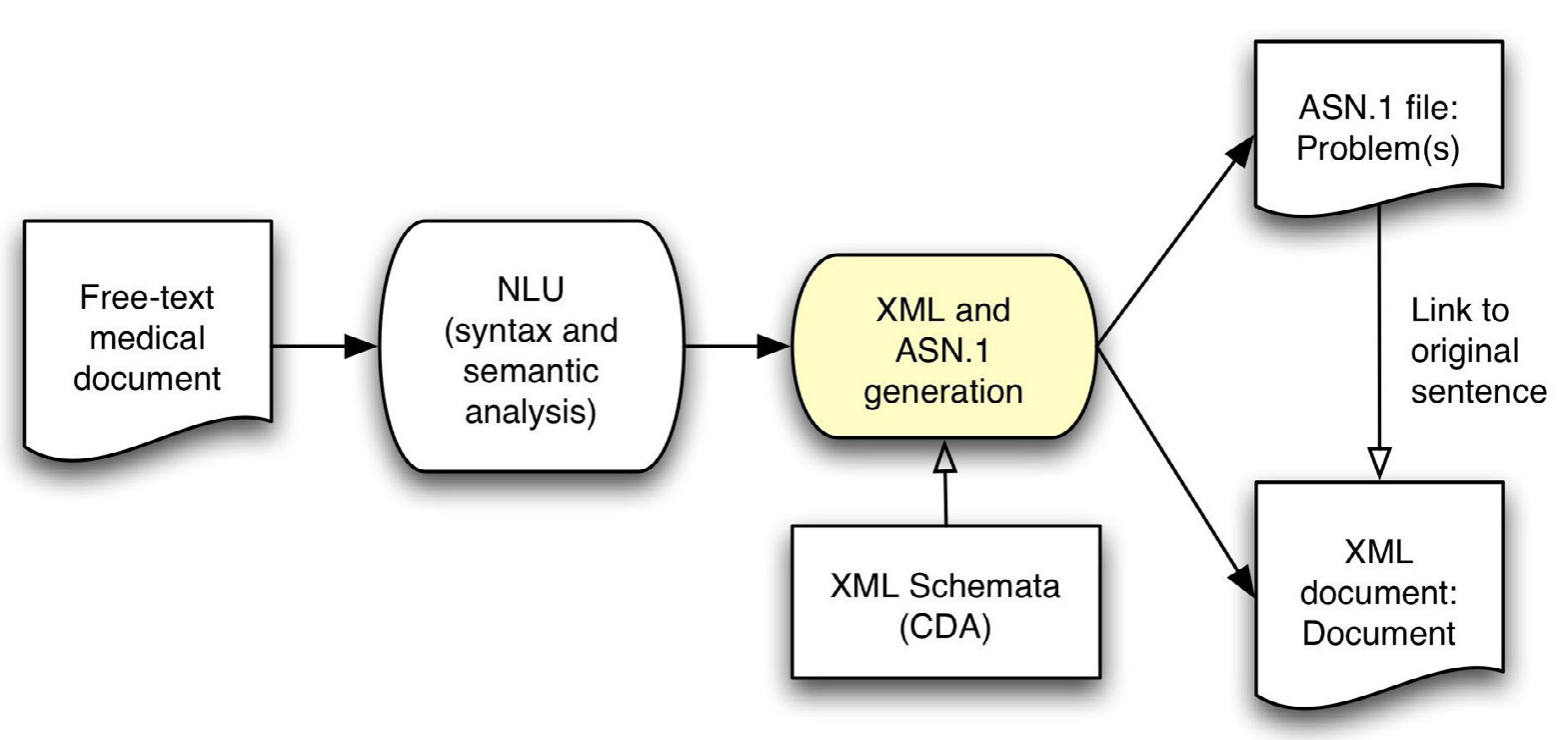
**Information model diagram. **Analysis of free-text documents results in the creation of a CDA version of the analyzed document and of an ASN.1 Problem record for each medical problem detected.

Medical documents are represented using HL7's Clinical Document Architecture (CDA) standard [69], with detected medical problems coded as Observations. CDA XML Schemata are therefore used for validation (Figure 1). The XML format allows us to link the extracted medical problem back to its source sentence(s): We use it to display the document(s) a medical problem was extracted from, with the sentence(s) containing mentions of the problem highlighted for easier reading. Medical problems are represented using an information model currently associated with the Clinical Information System in use at our institution: the *Clinical Event *model [79], instead of the custom model implemented in XML and described in a previous publication [68]. The *Clinical Event *model is implemented in ASN.1 [80].

### Background application

**Figure 2:**
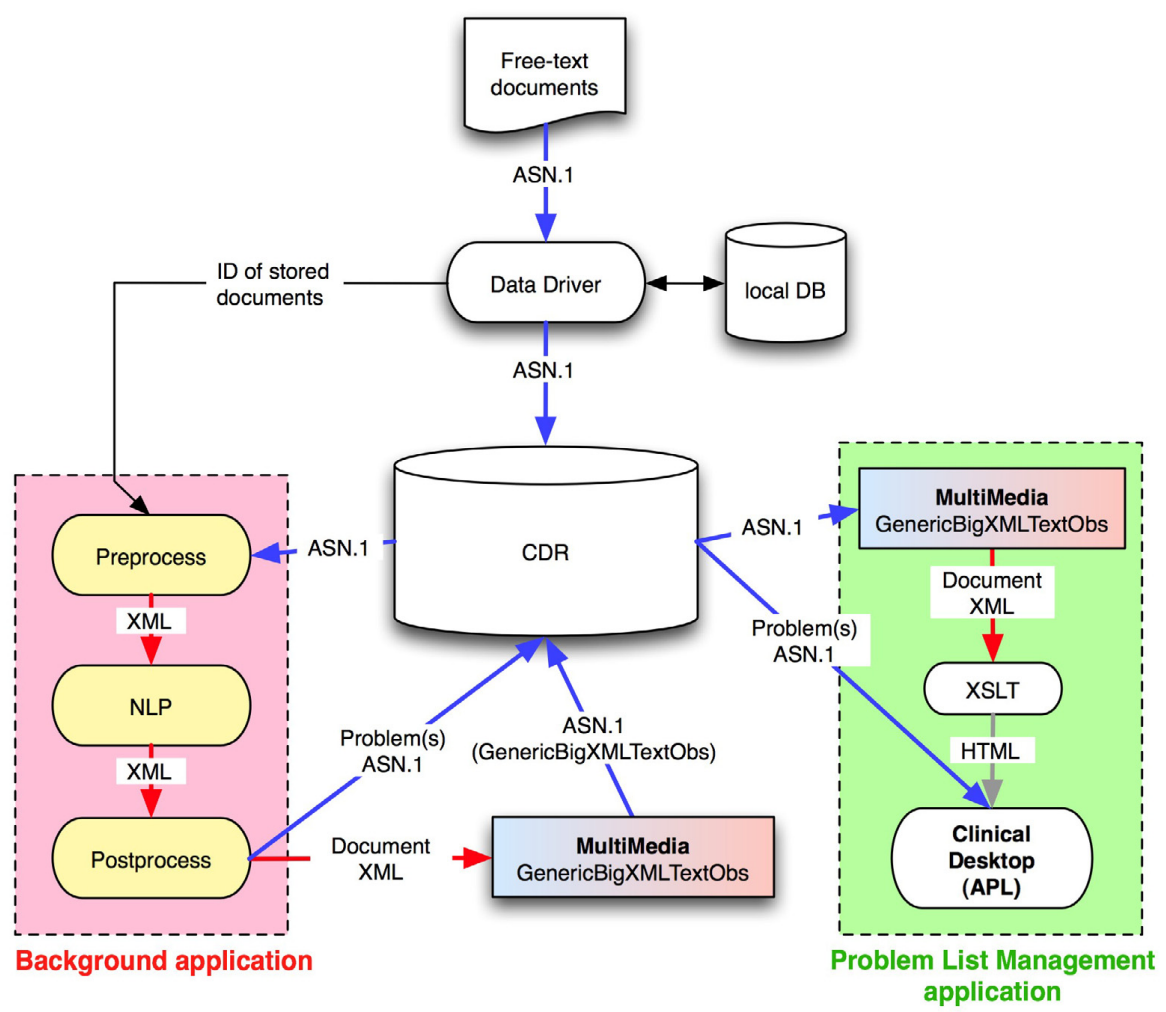
**Automated Problem List system diagram. **The two main components of the Automated Problem List system (the background application and the problem list management application) are displayed with the elements of our clinical information system they interact with. The ASN.1 data model used is called MultiMedia, with a GenericBigXMLTextObs data type. It allows storage of XML files as simple text but recognized as XML.

The application for processing clinical documents runs in the background and follows the sequence of steps depicted in Figure 2 and explained below.

In our organization, all data stored in the central database (CDR) pass through an event monitor called the "data driver". This tool recognizes medical events that may require additional processing and allows applications to subscribe to these events and to receive notification of their arrival and storage in the database. A subscription was created to route notification of free-text clinical documents to our system. The notification includes related information such as the patient identifier. We use the patient identifier to determine whether the patient is in the targeted group of cardiovascular patients. If this is the case, the corresponding document is retrieved from the CDR. The document processing phase then begins, starting with section and sentence detection.

#### Section detection

Sections are main paragraphs of the document, sharing a common class of information, like the "History of present illness", the "Physical examination", the "Family history", and many others. To develop this processing, we analyzed 200 documents randomly selected from a set of 5271 documents from cardiovascular adult inpatients at the LDS Hospital in 2002. Section titles were recognized using specific regular expressions. For example, in some "Progress Notes", section titles start with an uppercase letter after a carriage return and a space, and are followed by two colons. In some "Surgery Reports", all section titles are in uppercase letters, following a carriage return, and ending with a colon. The resulting section detection algorithm was implemented using regular expressions, and first used on this set of 5271 medical text documents to extract all possible section titles. 539 different titles were recognized and manually mapped to a list of 20 generic section titles. This latter step was required because we wished to use a version of the NLP module designed to integrate the title of a section as context in recognizing medical problems.

#### Sentence detection

After a section title is detected, the text following it until the next section title is extracted as the section's textual content. This text is then split into sentences using a sentence detection algorithm also implemented using regular expressions. The same 200 documents mentioned above and generic sentence boundary information described in another publication [81] were used to define the required regular expressions.

Before applying the sentence detection algorithm, some data cleaning was needed: white spaces (space, tabs, carriage return, etc.) before the first letter or number character and after the last one were removed. The regular expressions were then used to split the section text into sentences. Text was split at periods, exclamation and interrogation marks, and at white spaces, when those were preceded and followed by some specified characters, as in the following example:

(?<=[0-9a-z\.][0-9a-z%"])\.(?=[ \n\r]+[A-Z0-9])

This regular expression splits text at periods preceded by one number (0–9), lowercase letter (a-z), or period (.), this character being followed by one number, lowercase letter, percent sign (%), or quote ("). The period must be followed by one to many spaces or carriage returns, the latter being followed by one uppercase letter (A-Z) or number. For all twelve regular expressions used, see [Additional file 1].

A small pilot evaluation of our section and sentence detection algorithms was executed to determine their effectiveness in the clinical text documents planned for our subsequent studies. For this small evaluation, 20 documents from the test set described above were randomly selected, and sections and sentences were determined by the first author to build the reference standard. Each document was analyzed three times, in different random order, and at an interval of at least 3 days. Sections and sentences determined by the majority of the three reviews were finally used as reference standard. The section and sentence detection algorithms were then applied to these documents and results compared with the reference standard.

#### Natural language processing module

After the algorithms described above split the document's text into sections and sentences, each sentence is passed to the NLP module with context information including the document type and the section descriptor. The NLP module then analyzes each sentence and extracts potential medical problems, inferring the state of the problem using this contextual information. For example, a problem found in the Family History section will be stated as absent in the patient, if not found in another section of the document. At the sentence level, priority is given to the state present if the same problem is found more than once in the sentence. Thus, if the same problem is found once absent and once present in the same sentence, it is categorized as present at this sentence level. The priority of the state present is justified by examples like "the patient is known for *angina*, but hasn't suffered from *anginal pain *for more than two years". The first mention of the problem is stated as present, and the second as absent, and it will finally be considered present, since mention of this problem in the problem list with an *inactive *status is desirable.

At the document level, priority is given to the state absent. This means that if the same problem is found present in one sentence and absent in another, it will finally be categorized as absent in the document, unless the absent problem reference was found in the Family History section. Medical problems are often mentioned more than once in a document, and their states usually match, but examples of multiple mentions of the same problem with different states are common in documents like Discharge Summaries. A problem could be mentioned in the patient medical history, and then negated at the end of the document, when explaining that the problem was successfully treated during the hospitalization and is now absent. In this case, no mention of the problem in the problem list is desirable (considering that this type of document is analyzed by our system after discharge of the patient).

**Figure 3:**
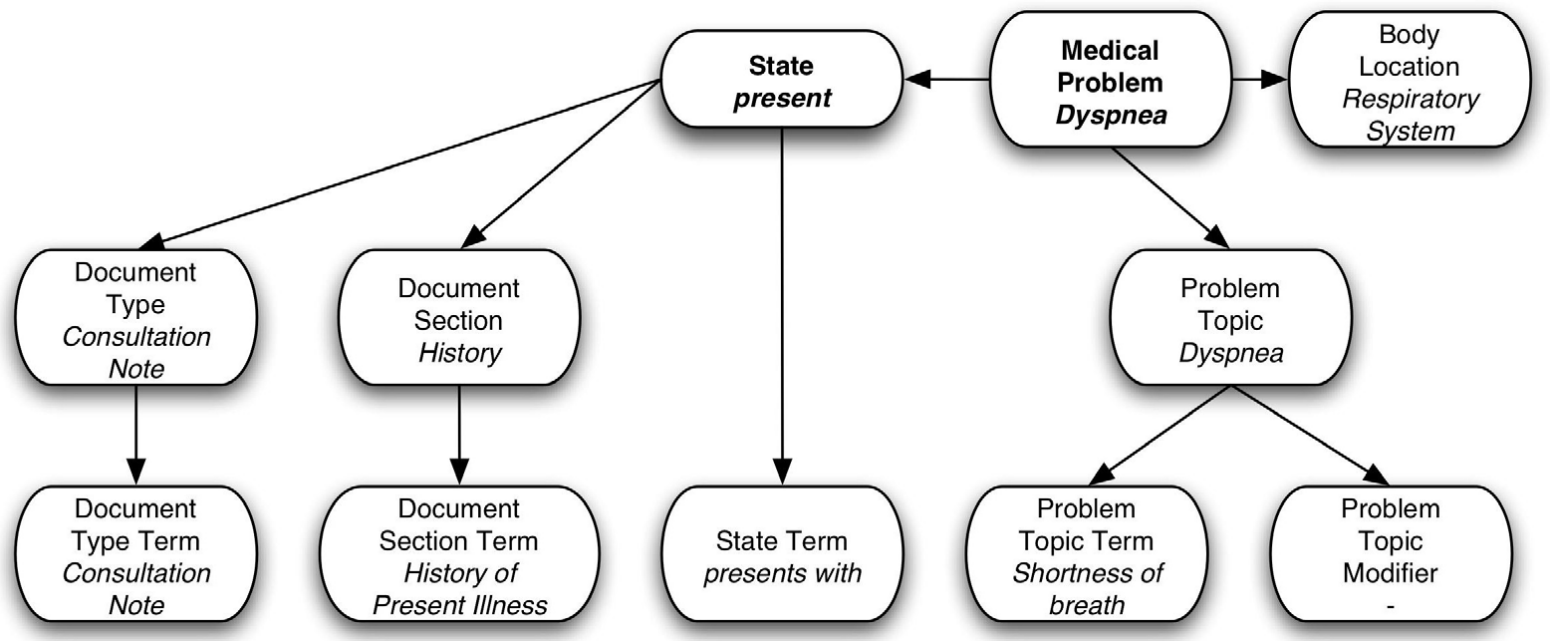
**Medical problems Bayesian Network. **Bayesian Network with example values for each node when analyzing the sentence "The patient presents with shortness of breath" in the "History of Present Illness" section of a "Consultation Note". Note the application of within-document context represented by the *Document Type *and *Document Section *nodes.

The NLP module was developed in two different versions. The first version was based on the NLP application developed locally and called SymText. This application is designed to do syntactic and semantic analysis, the latter using Bayesian Networks. It is trainable for different contexts and the semantic part was adapted to accommodate the clinical documents and medical concepts necessary to identify medical problems (e.g. history and physical, surgery report, consultation note, etc...). Training for this tool applies principally to the semantic model. Its semantics are represented as collections of Bayesian Networks representing the relationship between the words and phrases in a sentence and the concepts that they represent. Once the structure of the network is defined, the relationships among the semantic elements are captured as tables of probabilities. The Bayesian Network used by our application was made of 11 nodes and is displayed in Figure 3, with nodes corresponding to the word(s) or phrase(s) in the text at the bottom, and related concepts at the top.

**Figure 4:**
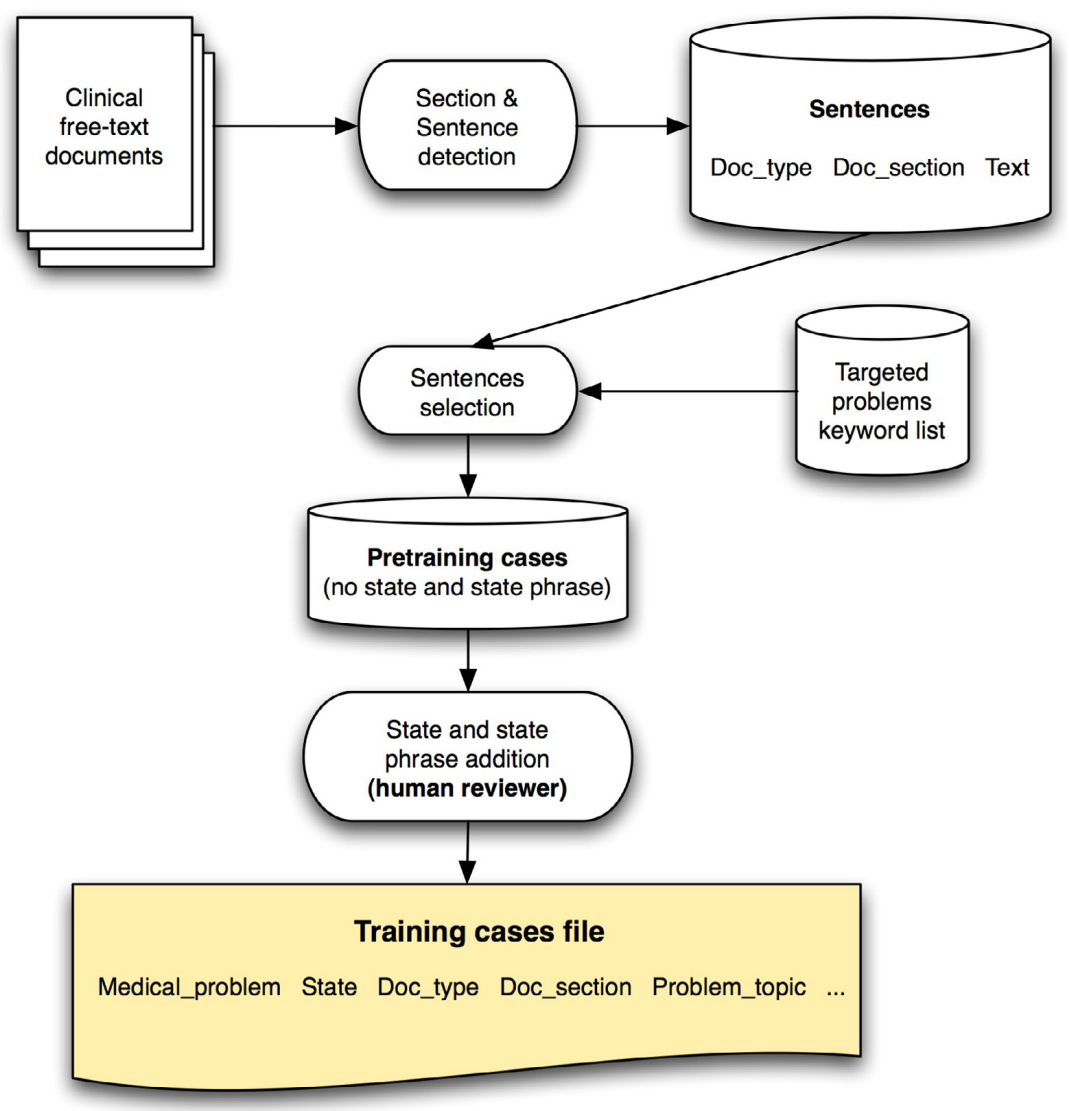
**Training cases creation process. **Sentences were first selected from the set of sentences resulting from the section and sentence detection of free-text documents. Regular expressions and a list of phrases representing possible ways of describing each of the 80 targeted problems were used for this task. The resulting pre-training cases were then augmented by a human reviewer adding the state and state phrase in each sentence. The resulting file contained 4436 training cases.

To capture these relationships, training cases are needed. We created those training cases using a semi-automated technique depicted in Figure 4. We started by applying the section and sentence detection algorithms on 3000 free-text documents and 91483 sentences were detected. Those documents were randomly selected from the set of 5271 test documents described above. Regular expressions and a list of phrases representing possible ways of describing each of the 80 targeted problems were then used to select sentences with mentions of the targeted problems. Lexical variants of phrases were partially taken into account by using regular expressions. The phrase table was built using the UMLS Metathesaurus MRCONSO table and a manually built table linking the 80 targeted problem concepts with all related subconcepts (e.g. *Right Bundle Branch Block *was linked to *Incomplete Right Bundle Branch Block*, *Complete Right Bundle Branch Block*, and *Other or unspecified Right Bundle Branch Block*). All phrases corresponding to the 80 concepts and their subconcepts were first retrieved from the MRCONSO table (6928 phrases). We then did some filtering, removing all phrases containing brackets, angled brackets, commas, forward slashes, squared brackets, dashes, and the words *NOS *or *unspecified *(e.g. "Anemia unspecified", "Anemia <1>", "Anemia (disorder)", "Anemia, aplastic", etc.). After removal of duplicates, the final table contained 4570 phrases.

Once this algorithm had selected target sentences, each sentence and its corresponding medical problem, along with the document type, the section title, and the sentence text were then proposed to a human reviewer (the first author). He proceeded to extract the word or phrase expressing the state of the problem, and added the state of the problem (present or absent) to the case. A medical problem was considered present if mentioned in the text as probable or certain in the present or the past (e.g. "the patient has asthma"; "past history positive for asthma"; "pulmonary edema is probable"), and considered absent if negated in the text or not mentioned at all (e.g. "this test excluded diabetes..."; "he denies any asthma"). The resulting file contained 4436 training cases. Training cases were prepared in a tab-delimited format as required by for training by Netica [82], the Bayesian Networks processing application used by SymText. A fine-tuning phase followed, using the NLP module to analyze sentences and eventually add training cases to improve the application's accuracy.

After the training, the tables in the NLP application's Bayesian Networks contained a statistical representation of the relationship between the words and phrases in the sentences and the medical problems that we had identified for extraction.

The second version of the NLP module was based on MetaMap Transfer (MMTx) [83], the Java version of MetaMap, and on the negation detection algorithm called NegEx [39]. This version worked in two steps. The first step used MMTx with the default full Metathesaurus data set to detect medical problems. The second step then used NegEx to infer the state of the concept detected, determining whether the concept was absent (negated) or present.

#### CDA document creation

**Figure 5:**
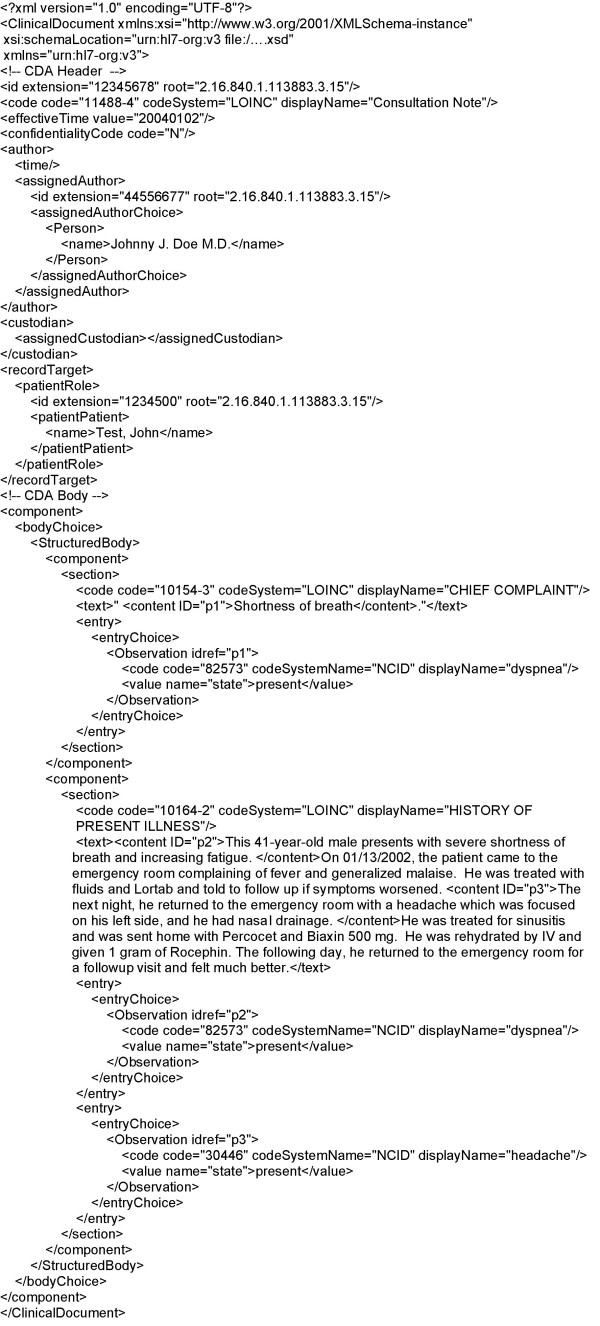
**Example CDA document. **XML Clinical Document Architecture version of the analyzed document.

**Figure 6:**
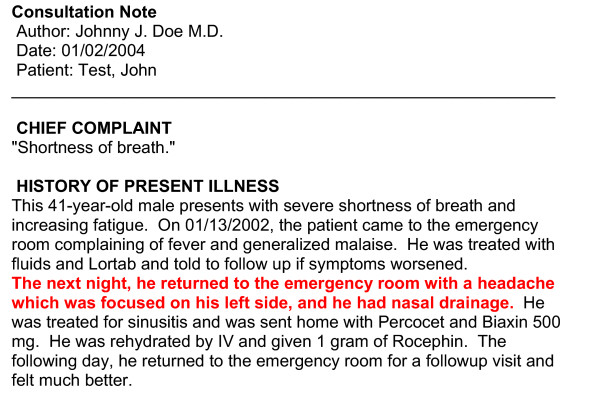
**Example rendered HTML version of the document. **Example of the customized HTML version of the document, as seen if linked from the problem *headache*

In addition to detecting problems referenced in medical documents, the system formats the documents according to the CDA standard. After section and sentence detection and NLP analysis, this XML-based CDA version of the source document is created, with each medical problem embedded as an encoded Observation, and with markup of the source sentences to allow the creation of the customized view of the document when a problem list user views the source documents associated with a problem. An example of a CDA document in both its XML form and its customized and rendered HTML (Hypertext Markup Language) form is displayed in Figures 5 and 6.

#### Document and problems storage

To avoid displaying repeated instances of a previously recognized problem, a patient's problems that are already stored in the CDR are analyzed. If the same problem has already been previously stored, and if this problem is not of "Family History" type, then the medical problem found by our system will not be stored in the CDR. In all other cases, the problem will be stored with a reference to the CDA version of the source documents. This link is required to allow recovery of this source document for display to users in the problem list management application. Finally, the medical problems recognized by the system as present are stored in the CDR, along with the resulting CDA document. These are given the status of "proposed" to distinguish them from problems stored manually in the CDR.

### Problem list management application

The problem list management application is the users' interface to the Automated Problem List system. It is the window through which end users interact with the medical problems proposed by the Automated Problem List system described above. For this project, an earlier problem list management application was rewritten to take advantage of the proposed problems. This new application for managing the problem list includes additional functionality focused on the proposed medical problems. It is designed to prompt the user to consider adding these extracted problems into the problem list.

Our clinical information system's user interface, called the Clinical Desktop, offers secured access to clinical data through specialized modules like the "Patient search", "Labs", "Medications", or "Problems" module.

The "Problems" module is our problem list management application. Features already present allow viewing, modifying, and adding medical problems in the problem list. An "infobutton" also allows access to medical knowledge relevant to the problem listed [78]. Filters control the display of medical problems based on their status (active, inactive, resolved, error, or proposed) and other personal preferences.

**Figure 7:**
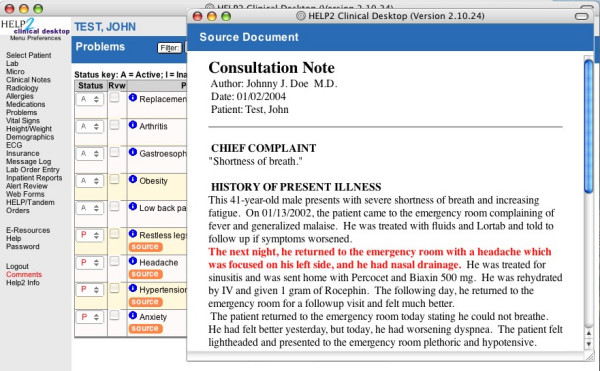
**Screenshot of the problem list management application. **Problem list management application with the viewer window showing the source document of the problem *headache *with the source sentence highlighted in red.

Functions added to take advantage of the proposed problems include the capability to list these problems with a new "proposed" status, and the provision of a link back to the source document to allow viewing of the document from which the problem was extracted. Human intervention is required to officially add a medical problem to the problem list, but for problems mentioned in the clinical documentation, addition is guided and simplified by this extended interface. To accept a problem, the user simply changes the status of the problem from "proposed" to "active" or "inactive", and to reject it, changes the status to "error". A "source button" has been added to each proposed problem listed, giving access to a viewer pop-up window displaying the source document with the source sentences of the medical problem highlighted in red (Figure 7). The user can locate the sentences and read them in a few seconds to determine whether the automatic system correctly proposed a problem for addition. To this end, the CDA version of the source document is retrieved from the CDR and transformed to a customized HTML version of the document. After preprocessing, an XSLT (XML Stylesheet Transformation) [84] stylesheet can be used to generate the HTML version of the document.

**Figure 8:**
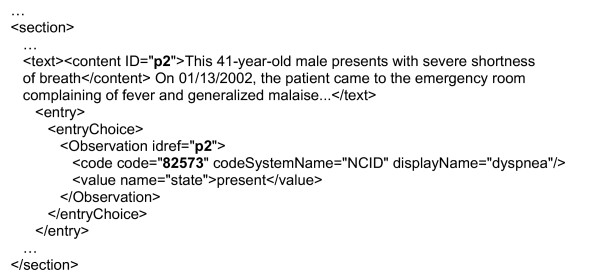
**Preliminary XML manipulation example. **In this extract of a CDA document, the code of the Observation element (*dyspnea *problem) and the reference identifiers are in bold characters. Reference identifiers link Observation elements (i.e. coded problems) to content elements (i.e. sentence(s) they were extracted from).

Preliminary processing of the XML document is required due to some missing features in XSLT, the most striking being an inability to change variables' values after their declaration and instantiation. This latter feature is needed to link the encoded *Observation *elements with the corresponding *content *elements in CDA documents. This preliminary phase therefore uses some more advanced XML manipulation features provided by a library for working with XML and XSLT on the Java™ platform. It searches the CDA document for *Observation *elements with codes corresponding to the medical problem's code. If *Observation *element(s) are found, corresponding *content *elements in the *text *element of the same section are searched and their tag name changed from *content *to *bold *when found, as described in Figure 8. The next step is the XSLT transformation, using the stylesheet included as [Additional file 2]. The whole retrieval and transformation process is fast, taking less than a second to retrieve, transform, and display the source document.

## Results

### Targeted medical problem set coverage

To estimate the proportion of coded diagnoses covered by our set of 80 targeted problems, all unique ICD-9-CM codes assigned to cardiovascular adult inpatients during 2003 in our institution (LDS Hospital, Salt Lake City, Utah) were retrieved. A total of 1531 different codes had been used. Our set of 80 targeted problems therefore covered only 5.2% of all possible codes. In the data set we found a total of 24160 ICD-9-CM code instances, and 15449 of them corresponded to one of our 80 targeted problems. Our set of problems therefore covered 63.9% of all code instances.

### Section and sentence detection

A small pilot evaluation of the section and sentence detection algorithms showed good performance. The section detection algorithm reached a sensitivity of 1.00 and a positive predictive value of 1.00. It detected all 146 sections present in the 20 randomly selected documents from our dataset, and detected only those 146 sections. With the sentence detection algorithm, a sensitivity of 0.889 (95% confidence interval 0.78-0.998) and a positive predictive value of 0.946 (0.907-0.985) were measured. 687 out of 731 sentences present in the test set of 20 documents were detected. Of those 687 sentences, 662 were correctly detected (i.e. true positives), and 25 were false positives.

## Discussion

The development and functions of the Automated Problem List system have been reported in this paper, along with results of two preliminary evaluations: The coverage of the set of 80 targeted problems was only about 5% of all possible diagnoses coded in ICD-9-CM, but about 64% of all instances of these coded diagnoses. The section and sentence detection algorithms performed well, with a sensitivity and positive predictive value of 100% when detecting sections, and a sensitivity of 89% and a positive predictive value of 94% when detecting sentences.

Some potential issues concerning this system have to be discussed. A key issue is the level of use of the problem list. Our goal is a system that eases the effort of maintaining an accurate, up-to-date problem list. However, our system can only be beneficial if users feel a commitment to maintain a problem list.

In the environment represented by the electronic medical record, there are much improved incentives to do so. Electronic implementation helps to mitigate key reasons for a lack of proper problem list maintenance. With the list available in one place, the EMR, rather than independently in each of the sites where the patient receives care, there is a significant incentive to both maintain and consult this document.

Another issue could be insufficient accuracy in the NLP portion of our system, with deficient sensitivity, positive predictive value, or speed. A sufficient sensitivity is required to significantly improve the quality of the problem list. We are seeking a sensitivity higher than 80%. A sufficient positive predictive value is also desirable to avoid overloading the collection of proposed problems with false positives. Those incorrectly proposed medical problems could make the use of the APL slower by requiring the user to reject an excessive number of incorrectly proposed problems. We are seeking a positive predictive value higher than 60%.

A last issue, which might effect our evaluation of this system, would be speed with which proposed medical problems are returned from the target documents. Our focus here is on the speed of the NLP module. This is clearly the slowest part of our system, requiring heavy computing power to allow deep text analysis. The evaluation of the NLP module will allow us to select the version to use (i.e. based on SymText or on MMTx/NegEx) for subsequent use and evaluation in a clinical setting. We will continue to study the issues of speed, along with scalability and accuracy as we evolve the system to manage problems beyond the cardiovascular domain.

In the future, the NLP module will be evaluated first, and, if satisfactory, the system described above will be evaluated in a clinical setting to determine its effectiveness in guaranteeing the accuracy, completeness, and timeliness of the medical problem list. We expect an increased proportion of correct problems, a reduced proportion of incorrect problems, and a reduced time between problem identification and addition to the problem list.

Coverage of instances of problem mentions in the free-text medical documents by the set of UMLS-derived phrases described in the Methods section could affect the accuracy of the NLP module, but this will not be evaluated in the future. We will focus on the bottom line question: performance of the NLP module when detecting medical problems.

The proposed Automated Problem List will be beneficial for many reasons: A better problem list should improve patient outcomes and reduce costs by reducing omissions and delays, improving the organization of care, and reducing adverse events. It will enhance decision-support for applications that require knowledge of patient medical problems to function optimally. A timely and accurate problem list should improve patient safety, an important and timely issue that has received substantial attention since the 1999 Institute of Medicine report [85].

## Conclusion

We have developed an Automated Problem List management system using NLP to extract potential medical problems from free-text documents in a patient's EMR. This system's goal is to improve the problem list's quality by increasing its completeness, accuracy and timeliness. By encouraging the use of a problem list of good quality, this system could potentially improve patient outcomes and security, improve the organization of care, reduce costs, and reduce adverse events.

The medical problem list figures prominently in our plans for computerized physician order entry and medical documentation in the new Clinical Information System (HELP2) currently under development at IHC. A well-maintained list will significantly enhance HELP2's applications. We believe that this clinical tool, which has been taught in medical schools and used sporadically in medical practice for decades, will become a key component for managing clinical information in systems that are developed to provide a longitudinal view of the health record.

## Competing interests

The author(s) declare that they have no competing interests.

## Authors' contributions

SM has conceived the Automated Problem List, developed it with the help of some acknowledged programmers, and implemented it. SM also has drafted this manuscript. PJH has proposed the general design and aim of the project, has guided its development and implementation, and has critically revised this manuscript. All authors read and approved the final manuscript.

## Pre-publication history

The pre-publication history for this paper can be accessed here:



## Supplementary Material

Additional File 1 **Sentence detection algorithm regular expressions **Text file listing all regular expressions used for sentence detection. Click here for file

Additional File 2 **XSLT stylesheet **Stylesheet used to transform the CDA version of the medical problem source document into its customized HTML version for viewing in the problem list management application. Click here for file
